# KCTD12 Regulates Colorectal Cancer Cell Stemness through the ERK Pathway

**DOI:** 10.1038/srep20460

**Published:** 2016-02-05

**Authors:** Liping Li, Tingmei Duan, Xin Wang, Ru-Hua Zhang, Meifang Zhang, Suihai Wang, Fen Wang, Yuanzhong Wu, Haojie Huang, Tiebang Kang

**Affiliations:** 1 State Key Laboratory of Oncology in South China, Sun Yat-Sen University Cancer Center, Guangzhou 510060, China; 2 School of Biotechnology, Southern Medical University, Guangzhou 510515, China; 3 Department of Pathology, First Affiliated Hospital of Sun Yat-sen University, Guangzhou 510080, China; 4 Department of Biochemistry and Molecular Biology, Mayo Clinic College of Medicine, Rochester, MN 55905, USA

## Abstract

Targeting cancer stem cells (CSCs) in colorectal cancer (CRC) remains a difficult problem, as the regulation of CSCs in CRC is poorly understood. Here we demonstrated that KCTD12, potassium channel tetramerization domain containing 12, is down-regulated in the CSC-like cells of CRC. The silencing of endogenous KCTD12 and the overexpression of ectopic KCTD12 dramatically enhances and represses CRC cell stemness, respectively, as assessed *in vitro* and *in vivo* using a colony formation assay, a spheroid formation assay and a xenograft tumor model. Mechanistically, KCTD12 suppresses CRC cell stemness markers, such as CD44, CD133 and CD29, by inhibiting the ERK pathway, as the ERK1/2 inhibitor U0126 abolishes the increase in expression of CRC cell stemness markers induced by the down-regulation of KCTD12. Indeed, a decreased level of KCTD12 is detected in CRC tissues compared with their adjacent normal tissues and is an independent prognostic factor for poor overall and disease free survival in patients with CRC (*p* = 0.007). Taken together, this report reveals that KCTD12 is a novel regulator of CRC cell stemness and may serve as a novel prognostic marker and therapeutic target for patients with CRC.

Colorectal carcinoma (CRC) remains one of the most aggressive cancers in the world. Every year, more than 1.2 million patients with CRC are diagnosed, and almost 50% die from the disease. Surgery, radiotherapy and chemotherapy are still the predominant therapeutic strategies^1^. Although surgery combined with chemoradiotherapy represents a viable treatment option for early stage tumors, the majority of patients are not diagnosed until the late stage, for which the 5 year survival rate post-surgery decreased from 69.2% to 11.7%^2^.

Cancer stem cells (CSCs) are considered to be responsible for recurrence and metastasis during CRC tumorigenesis^3^. CSCs, possessing self-renewal characteristics, initiate tumor growth and promote chemotherapy and radiation resistance, which are considered to be responsible for CRC progression and recurrence^4^,^5^. Targeting the determinants of CRC cell stemness has been proposed as a therapeutic strategy^6^.

KCTD12 (potassium channel tetramerization domain containing 12, pfetin), which contains a voltage-gated potassium (K^+^) channel tetramerization T1 domain and a BTB/POZ (Bric-a-brac, Tram-track, Broad complex poxvirus and zinc finger) domain, belongs to the KCTD family and was initially identified in cochlea. In addition to being a K+ channel protein that responds to membrane potential^7^, KCTD12 also acts as an auxiliary subunit of GABAB (γ-aminobutyric acid type B) receptors, which regulate emotionality and neuronal excitability^8^,^9^. Interestingly, high KCTD12 expression indicates a favorable prognosis and could act as an independent prognostic factor for GIST (gastrointestinal stromal tumors)^10^, most likely due to the control of tumor and tumor stem cell proliferation by GABA signaling^11^. In addition, other KCTD family members, such as KCTD21, 11, and 6, have been shown to regulate the growth of MB (medulloblastoma) stem cells through the histone deacetylase HDAC1 and Hh/Gli^12^,^13^,^14^. However, there is no information about whether KCTD family members play crucial roles in CRC cell stemness. As described here, using HT29 cells and their spheroids, we observed that KCTD12 was the most altered member of the KCTD family in the spheroids of HT29 cells, leading to our speculation that KCTD12 plays a crucial role in CRC cell stemness. In verification of this hypothesis, our data support that KCTD12 is a potential regulator of CRC cell stemness at a cellular level, in an animal model and in clinical samples.

## Results

### KCTD12 is down-regulated in the spheroids of HT29 cells

**Figure 1:**
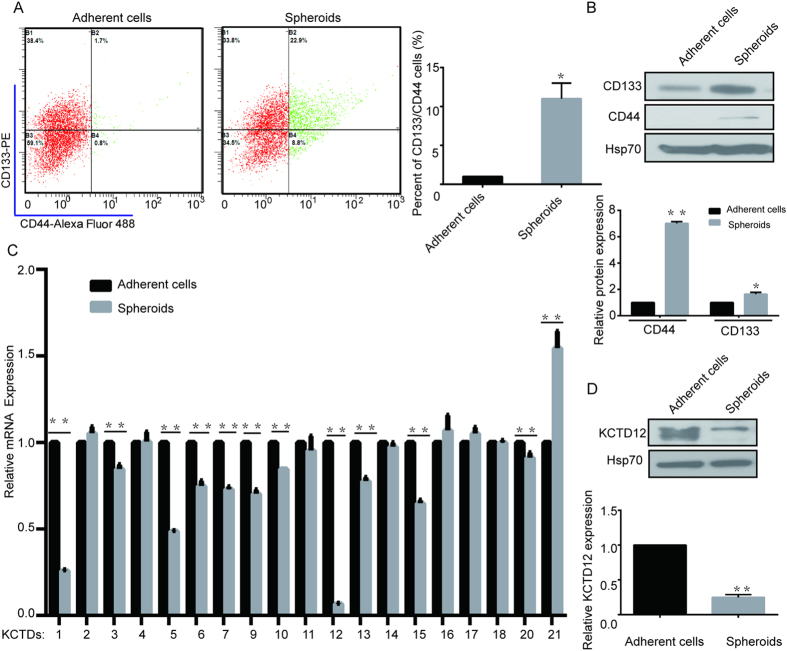
KCTD12 is down-regulated in CSC-like HT29 cells. (**A**) Representative flow cytometry plots and quantitative analysis showing the percentages of CD44^+^ and CD133^+^ cells in normal adherent and spheroid cultures of HT29 cells. (**B**) CD44 and CD133 expressions were analyzed by Western blotting in adherent and spheroid cultures of HT29 cells. (**C**) Quantitative real time PCR analysis of the relative mRNA levels of the KCTD family members in adherent and spheroid cultures of HT29 cells. (**D**) KCTD12 expression was analyzed by Western blotting in adherent and spheroid cultures of HT29 cells. The results are presented as the means ± SD, and all data are representative of three independent experiments. *P < 0.05, **P < 0.01.

To investigate whether KCTD family members are involved in the stemness of CRC cells, we first enriched for stemness characteristics of HT29 cells by culturing them as spheroids for 8 days^15^. As shown in Fig. 1A,B, the percentages of cells expressing CD133 and CD44, two well-known stemness markers, were dramatically increased as shown by flow cytometry assay and western blotting. Subsequently, the mRNA levels of KCTD family members were compared between the spheroids and the parental HT29 cells. As the results in Fig. 1C show, the mRNA levels of KCTD1, 5 and 12 were decreased, while the levels of KCTD21 were increased in spheroids of HT29 cells. Notably, KCTD12 was the most significantly decreased family member in spheroids of HT29 cells (Fig. 1C), which was further confirmed by the observation that the KCTD12 protein was also significantly decreased in the spheroids of HT29 cells (Fig. 1D). However, KCTD8 and KCTD19 could not be detected in HT29 cells. These findings indicate that KCTD12 may play a crucial role in the stemness of CRC cells.

### KCTD12 regulates CRC cell stemness in cell lines

**Figure 2:**
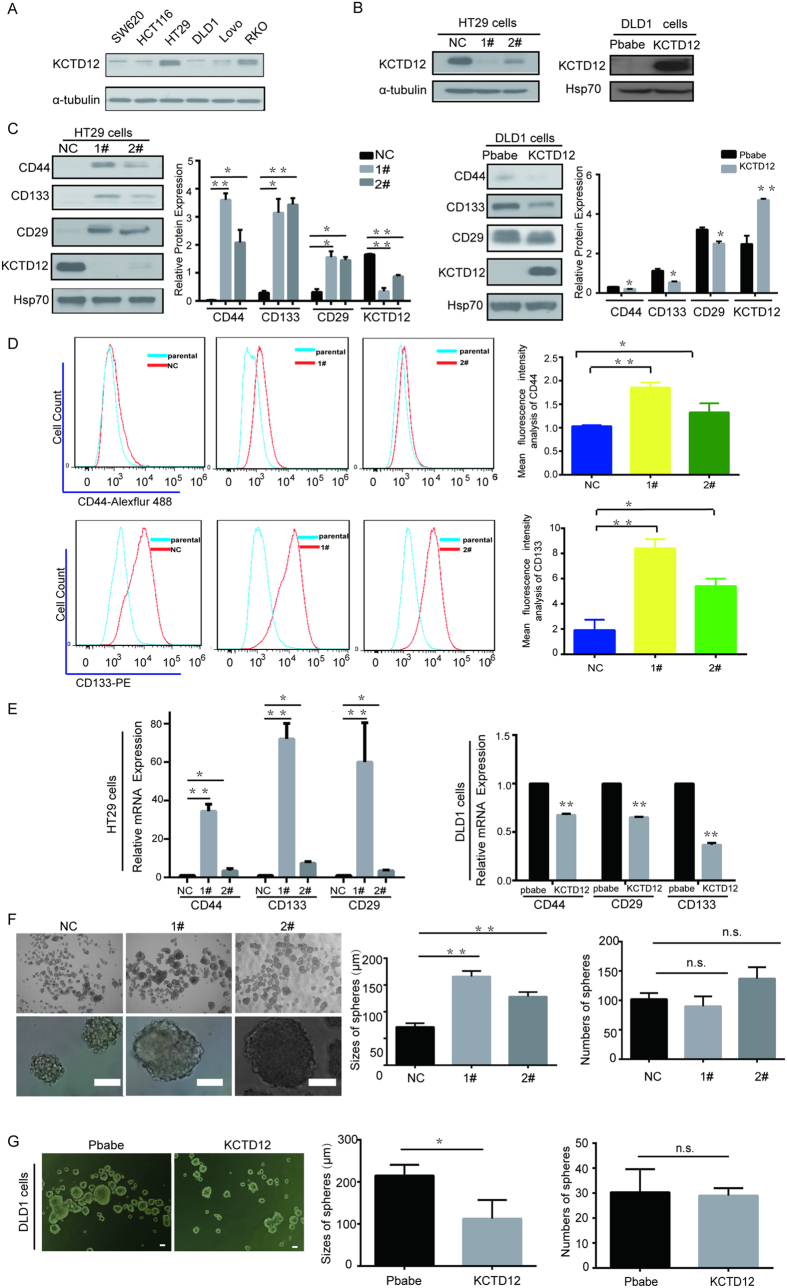
KCTD12 suppresses the stemness of CRC cells. (**A**) KCTD12 protein was analyzed by Western blotting in the indicated CRC cell lines. (**B**) The indicated stable cell lines with silencing or overexpression of KCTD12 were analyzed by Western blotting. α-tubulin or HSP70 was used as the loading control. (**C**-**E**) CD44, CD133 and CD29 levels were analyzed by Western blotting, qRT-PCR and flow cytometry, in the indicated stable cell lines. Red lines indicating the mean intensity of fluorescence of CD44^+^ or CD133^+^ were quantified by Flow-J software in the flow cytometry analysis. The mean intensity of fluorescence of CD44^+^ or CD133^+^ was calculated in triplicates. (**F**,**G**) Images and quantification of the number and size of spheres formed from the indicated stable cell lines in the absence of serum for 7 days. Original magnification in F, 40×(upper), 400×(lower). Original magnification in G, 40×. Scale bars, 100 μm. The results are presented as the means ± SD, and all data are representative of three independent experiments. *P < 0.05, **P < 0.01.

Next, we asked whether KCTD12 influences stemness using CRC cell lines with varying levels of KCTD12 (Fig. 2A). HT29 cells and DLD1 cells were chosen to knockdown and overexpress KCTD12, respectively (Fig. 2B). As shown in Fig. 2C, the silencing and the overexpression of KCTD12 were capable of increasing and decreasing, respectively, the well-known CRC cell stemness markers CD44, CD133 and CD29 at the protein and mRNA levels. Consistently, the percentages of cells positive for CD44 or CD133 were dramatically increased when KCTD12 was knocked down in HT29 cells (Fig. 2D). In addition, the silencing and the overexpression of KCTD12 in HT29 or DLD1 cells increased and decreased the sizes of spheres, respectively, while having no effect on the numbers of spheres (Fig. 2E,F). Taken together, these results indicate that KCTD12 is critical to the stemness of CRC cells.

### KCTD12 is involved in the self-renewal ability of CRC cells *in vitro* and in the tumorigenesis of CRC cells *in vivo*

**Figure 3:**
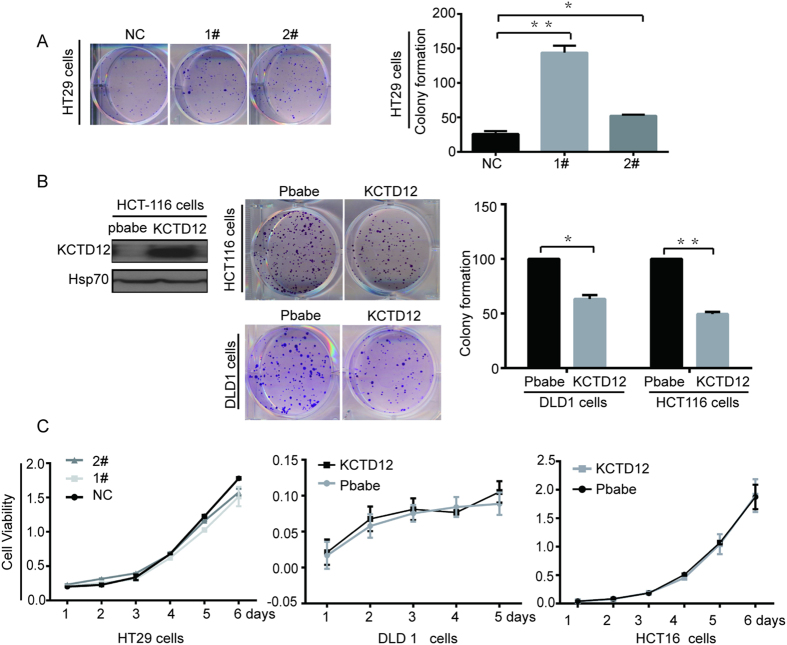
KCTD12 inhibits the colony formation of CRC cells *in vitro*. (**A,B**) The colony formation assays were performed in the indicated stable cell lines. (**C**) The cell proliferation was measured by MTT in the indicated stable cell lines. The results are presented as the means ± S.E. of three independent experiments. *P < 0.05, **P < 0.01.

**Figure 4:**
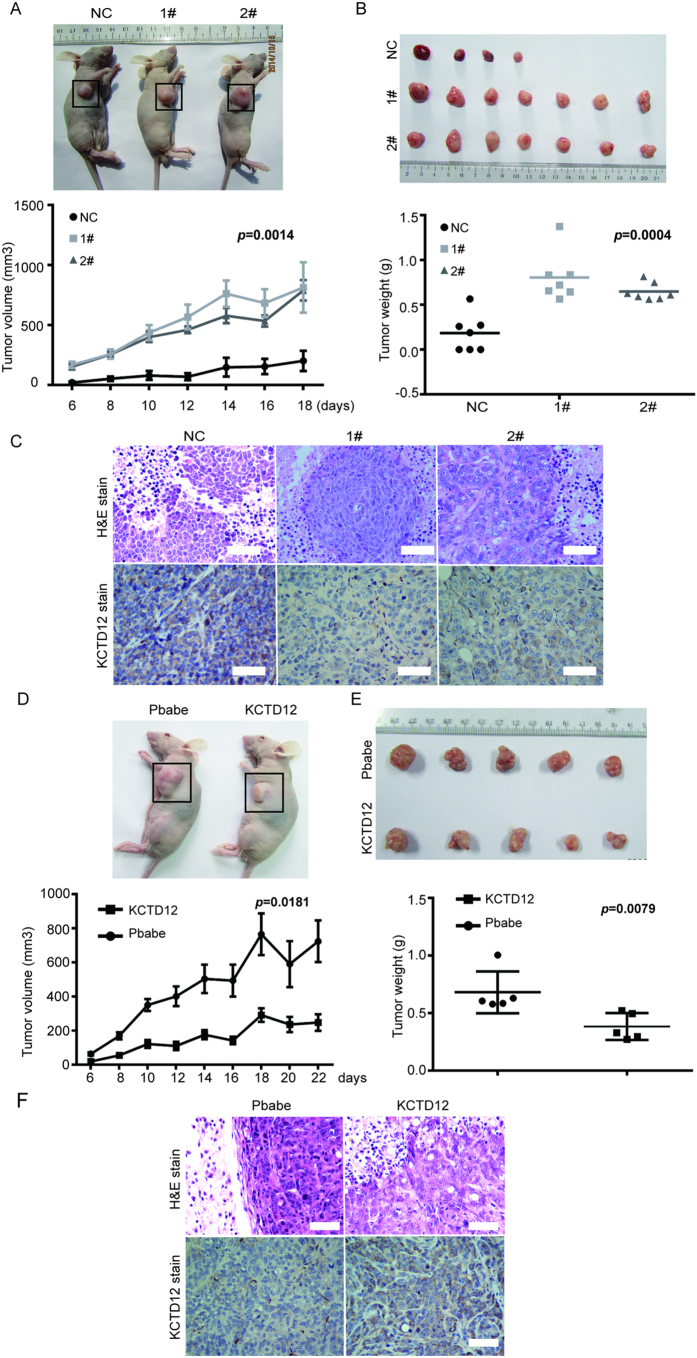
KCTD12 represses the tumorigenicity of CRC cells *in vivo*. (**A,B**) A xenograft model consisting of nude mice with HT29 cells harboring KCTD12 silencing injected into the armpits of 4 week old mice (n = 7/group). The images of mice harboring tumors (left) and tumors from the mice (right). Tumor volumes were measured every two days (left). Mean tumor weights were calculated. (**D,E**) A xenograft model consisting of nude mice with DLD1 cells overexpressing KCTD12 were injected into the armpits of 4 week old mice (n = 5/group). The images of mice harboring tumors (left) and tumors from the mice (right). Tumor volumes were measured every two days (left). Mean tumor weights were calculated. The results are presented as the means ± SD. *P < 0.05, **P < 0.01. (**C,F**) H&E staining of tumors and IHC staining for KCTD12 protein in these cells. Original magnification, 200×. Scale bars, 100 μm.

We further explored the functions of KCTD12 in the self-renewal and tumorigenesis of CRC cells. First, as shown in Fig. 3A by the colony formation assay, the knockdown of KCTD12 in HT29 cells significantly enhanced the cells’ colony formation capacity, whereas the overexpression of KCTD12 in both DLD1 and HCT116 cells reduced this capacity (Fig. 3B). However, the alteration of KCTD12 in these cells did not affect their proliferation (Fig. 3C). These results indicate that KCTD12 is involved in the self-renewal ability of CRC cells *in vitro*. Second, as shown in Fig. 4, the knockdown of KCTD12 in HT29 cells promoted, whereas the overexpression of KCTD12 in DLD1 cells inhibited, tumor growth in nude mice, as measured by tumor volumes and weights. These results suggest that KCTD12 plays a crucial role in CRC tumorigenesis *in vivo*.

### Silencing of KCTD12 enhances the drug resistance of CRC cells

**Figure 5:**
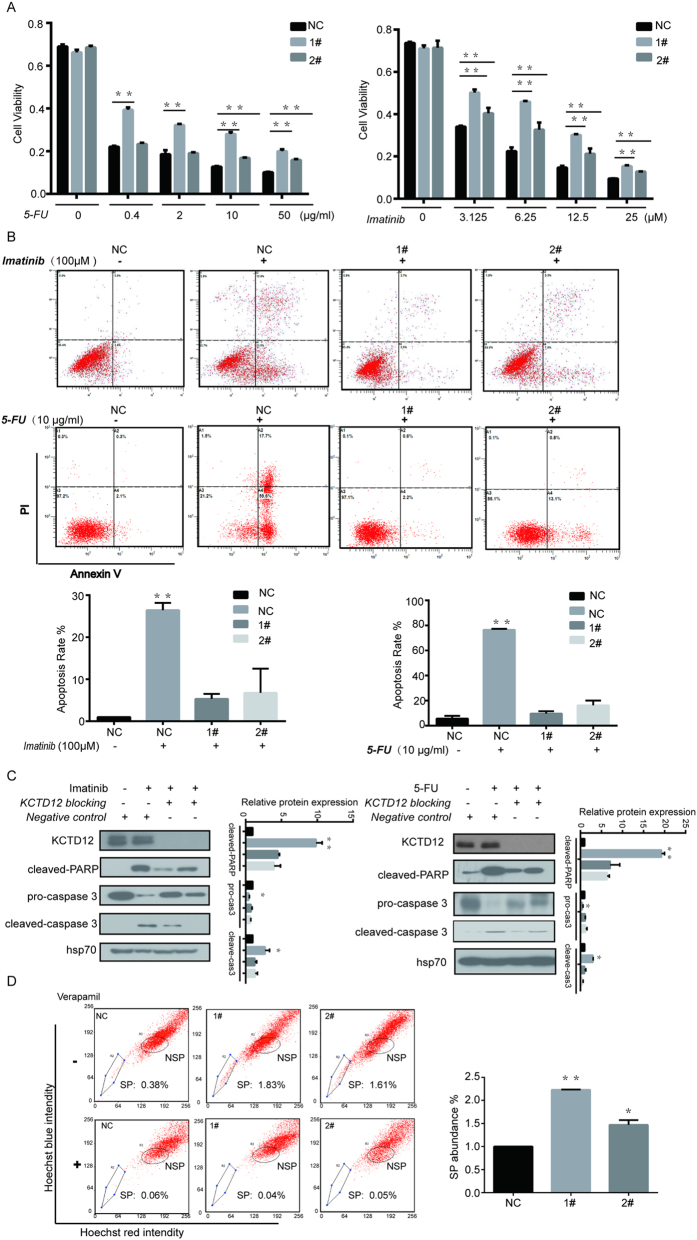
Silencing of KCTD12 enhances the drug resistance to both imatinib and 5-FU in HT29 cells. (**A**) HT29 cells with silenced KCTD12 were seeded in 96-well plates at a density of 5 × 10^3^ per well and treated with increasing concentrations of imatinib and 5-FU, as indicated. Cell viabilities were detected by the MTT assay. (**B**) HT29 cells with silenced KCTD12 were seeded in 6-well plates at a density of 5 × 10^5^ per well and treated with 100 μM imatinib for 24 h or 10 μg/ml 5-FU for 48 h, as indicated. The apoptosis rates were detected with the Annexin ν/PI kit and analyzed by flow cytometry. (**C**) Western blotting analysis of cleaved-PARP, procaspase3 and cleaved caspase 3. The experiments were repeated three times. (**D**) The side population (SP) cells assay. HT29 cells with silenced KCTD12 were treated with 50 μg/ml verapamil and 0.1 μg/ml Hoechst 33342 dye and subjected to flow cytometric analysis. Representative flow cytometric histograms demonstrating a distinct SP cells fractions. The quantified results were presented as the means ± SD (n = 3). *P < 0.05, **P < 0.01.

As chemoresistance is another characteristic of cancer stem cells, we then sought to test whether there is a correlation between KCTD12 level and drug resistance in CRC cells. KCTD12 is a prognostic biomarker for the treatment of GIST patients with imatinib mesylate^16^, and imatinib is a potential therapeutic strategy for patients with CRC^17^. We therefore chose to use both 5-FU, a commonly used therapeutic for patients with CRC, and imatinib mesylate to test our hypothesis. As shown in Fig. 5A, HT29 cells with KCTD12 knockdown displayed enhanced viability in the presence of varying concentrations of imatinib and 5-FU. Consistently, decreases of 20% and 60% in apoptosis rates were detected in the KCTD12 knockdown HT29 cells treated with 100 μM imatinib and 10 μg/ml 5-FU, respectively (Fig. 5B), results that were further supported by the cleavage of both PARP and procaspase 3 (Fig. 5C). Given that chemoresistance affords CSCs drug-exclusion properties, the side population (SP) cells using Hoechst-33342 dye were examined in KCTD12 knockdown HT29 cells. The results indicated that the down-regulation of KCTD12 dramatically increased the SP abundance (Fig. 5D). Collectively, the results suggest that the down-regulation of KCTD12 enhances the resistance of CRC cells to both imatinib and 5-FU.

### KCTD12 regulates CRC cell stemness via the ERK pathway

**Figure 6:**
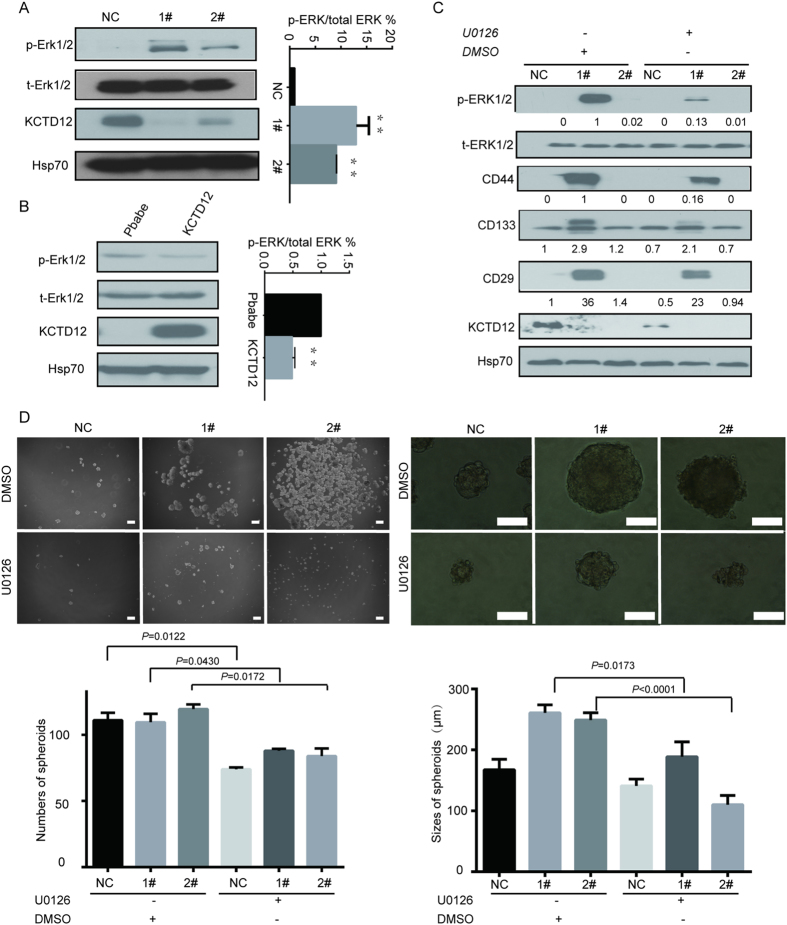
KCTD12 regulates stemness of CRC cells via the ERK signaling pathway. (**A,B**) Phosphorylation of ERK1/2 (p-ERK1/2) and total ERK1/2 (t-ERK1/2) were detected using western blotting in the indicated stable cell lines. (**C**) HT29 cells with silenced KCTD12 were treated with U0126 (30 μM) for 24 h. Western blotting was performed to detect t-ERK1/2, p-ERK1/2, CD44, CD133 and CD29. Hsp70 was used as a loading control. (**D**) The sphere formation assays were performed in HT29 cells with silenced KCTD12 and treated with U0126 or DMSO for 7 days. Images and quantification of the numbers and sizes of spheres formed were calculated. The experiments were repeated three times. *P < 0.05, **P < 0.01. Scale bars, 200 μm (left) and 100 μm (right).

Given that KCTD12 acts as a component of the GABA_B_ complex, downstream of which is the ERK pathway, we sought to determine whether the ERK pathway is involved in the KCTD12-mediated regulation of CRC cell stemness. As shown in Fig. 6A,B, phosph-ERK1/2 levels were dramatically increased in HT29 cells with silenced KCTD12 and decreased in DLD1 cells with overexpressed KCTD12. Moreover, U0126, an ERK 1/2 inhibitor, abrogated the increases in CD44, CD133 and CD29 levels in HT29 cells induced by the knockdown of KCTD12 (Fig. 6C). Likewise, the inhibition of the ERK pathway by U0126 reduced the sizes of spheres in KCTD12 knockdown HT29 cells (Fig. 6D). These results indicate that KCTD12 regulates CRC cell stemness via the ERK pathway.

### Low KCTD12 expression indicates a poor prognosis of patients with CRC

**Figure 7:**
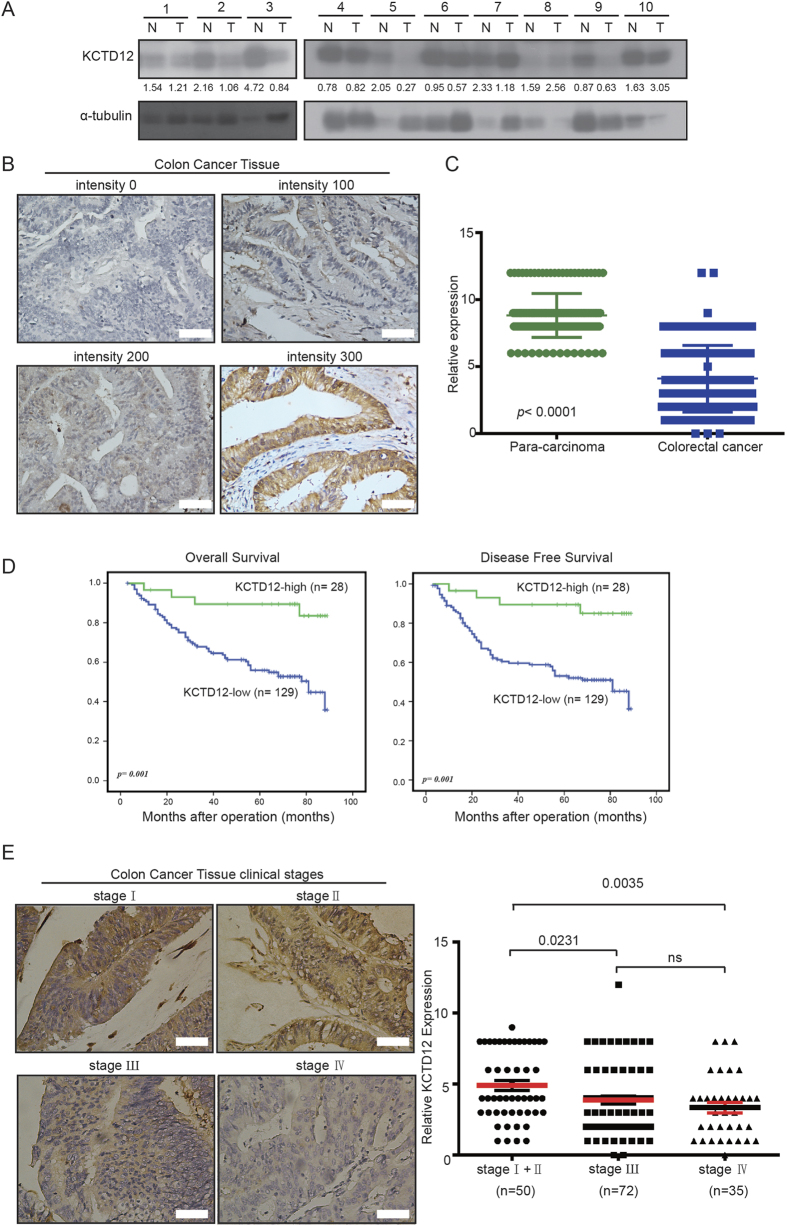
Low expression of KCTD12 was detected in human colorectal cancer tissues. (**A**) The KCTD12 protein levels in CRC tumor tissues (T) and their adjacent normal tissues (N) were analyzed by western blotting. (**B**) The KCTD12 protein levels in 157 CRC tissues were analyzed by IHC. The images represented differential staining intensities of KCTD12. (**C**) The relative KCTD12 expression levels were analyzed based on scores, and the results were shown as the means ± SD. (**D**) The correlation between KCTD12 expression and overall survival rate (*p* = 0.001) and disease free survival rate (*p* = 0.001) of CRC patients (n = 157) were determined using Kaplan-Meier survival and log-rank analysis. (**E**) The images represented differential KCTD12 staining intensities in different clinical stages (left). The correlation between KCTD12 expression and the clinical stage were analyzed between stageI/II, stage Ш and stage IV. Stage I and Stage II cases were combined into one group. *P < 0.05, **P < 0.01.

Finally, we analyzed the clinical relevance of KCTD12 in CRC samples. As shown in Fig. 7A, the protein level of KCTD12 was significantly higher in normal tissues than in CRC tumor tissues. Immunohistochemical (IHC) staining showed that KCTD12 was localized to the cytoplasm of CRC cells (Fig. 7B) and that its expression was significantly lower in CRC tissues compared with their adjacent normal tissues (*p* < 0.0001, Fig. 7C). To investigate the correlation between KCTD12 expression and the clinicopathological features of patients with CRC, the 157 patient samples were divided into two groups (high and low KCTD12) based on IHC density scores. The chi-square test revealed that the KCTD12 level was strongly related to the clinical stage (*p* = 0.027), tumor size (*p* = 0.021) and vital status (*p* = 0.003) (Table 1). As shown in Fig. 1D, Kaplan-Meier survival curves and the log-rank test showed that the KCTD12 expression level was significantly correlated with overall survival (OS) and disease free survival (DFS) of patients with CRC (*p* = 0.001). The univariate and multivariate analyses revealed that the KCTD12 expression level (*p* = 0.007), with a Hazard Ratio (HR) of 0.239 and a 95% CI of 0.084–0.677, was an independent prognostic factor in patients with CRC (Table 2). To examine the correlation between the KCTD12 expression level and CRC clinical stages, Stage I and Stage II cases were grouped together in our analysis due to a limited sample number. The results showed that the expression level of KCTD12 in stage I and II cases is higher than that in stage III and stage IV cases (*p* < 0.05, Fig. 7E). Taken together, these results indicate that KCTD12 may serve as a prognostic indicator for patients with CRC.

## Discussion

In this report, the down-regulation of KCTD12 is detected in colorectal CSC-like cells, and a low level of KCTD12 is associated with a poor prognosis of patients with CRC. Functionally, KCTD12 regulates CRC cell stemness characteristics, such as self-renewal, tumorigenesis and drug resistance, through the ERK pathway. This is the first report to reveal that KCTD12 regulates CRC cell stemness through the ERK pathway.

CRC stem cells have been proven to initiate tumorigenesis and recurrence of CRC during progression of the disease, although how to target CRC stem cells remains a problem in this field^6^. Although multiple molecules and signaling pathways have been shown to associate with the stemness of CRC cells, the regulatory mechanisms of CRC stem cells are not yet completely elucidated. In this report, we demonstrated that KCTD12 regulates CRC cell stemness, as the silencing and the overexpression of KCTD12 promotes and inhibits, respectively, the stemness characteristics of CRC cells, such as self-renewal, tumorigenesis and drug resistance *in vitro* and *in vivo*, most likely via an ERK-dependent mechanism. Consistently, a lower level of KCTD12 is a negative independent prognostic factor for patients with CRC. This information may be useful for patients with CRC, especially for those with a low level of KCTD12. Given that MAPK/ERK pathway activation has been linked to the regulation of CRC pathogenesis, progression and chemoresistance^18^, the combination of an ERK inhibitor, such as U0126, with imatinib and/or 5-FU would be beneficial to the treatment of certain CRC patients, as this combination would increase drug sensitivities.

CSCs represent a small subpopulation of cancer cells that are capable of maintaining self-renewal, survival and chemoresistance. To settle this problem in the context of traditional chemotherapeutics, many studies have shown that the combination of chemoresistance-related proteins and agents, such as the ABC transporter^19^, the multi-pass membrane protein SLC6A6^20^ and microRNAs^21^, with conventional chemotherapeutic drugs improve the efficacy of CRC treatment. The new chemotherapeutic combination (5-FU, irinotecan, and oxaliplatin) and monoclonal antibodies against the epidermal growth factor receptor prevented the metastasis of CRC and improved the survival of patients compared with what has been observed over the past decades^22^. Elevated Lgr5 levels promoted spheroid resistance to 5-FU and oxaliplatins^23^. It is clear that the discovery of more molecules regulating CSCs will improve therapeutic efficacy.

KCTD8, 12 and 16 are identified as components of the auxiliary GABA_B_ receptor subunits^24^, but only KCTD12 generates a desensitizing GABA_B_ receptor response^8^,^25^ and suppresses the proliferation of GIST cells via interference with GABA_B_ signaling^26^, which has been reported to play important roles in several human cancers, including CRC^27^,^28^. This notion is also supported by our finding that KCTD12 may be an important regulator of CRC cell stemness via the ERK pathway, a downstream component of GABA_B_ receptor signaling. Although not currently understood, we will further investigate how KCTD12 modulates the ERK pathway, and we speculate that the GABA_B_ signaling pathway may also be crucial for cell survival, tumorigenicity, and drug resistance in some cancers, including CRC, as previously shown in the literature^29^,^30^,^31^.

In summary, we have determined that KCTD12 plays an important role in the tumorigenesis of CRC progression via activation of the ERK signaling pathway and could serve as a useful biomarker for the prognosis of patients with CRC.

## Materials and Methods

### Human colorectal cancer clinical specimens

157 paraffin-embedded primary CRC tissues were obtained from CRC patients who were diagnosed and underwent primary tumor resections from 2000 to 2008 at the Sun Yat-Sen University Cancer Center, Guangzhou, China. The clinicopathological characteristics of the patients were summarized in Supplementary Table 1 and included the stages of the patients’ tumors: stage Ι (N = 12), stage II (N = 38), stage III (N = 72) and stage IV (N = 35). Ten paired CRC tissue specimens and the adjacent normal tissues were collected and stored at −80 °C immediately after surgery for the western blotting assay. All patients provided written informed consent for research purposes, according to guidelines approved by the institutional Review Board of Ethics at the Sun Yat-Sen University Cancer Center. The experimental protocols of all experiments involving human were approved by the ethical committee of Sun Yat-Sen University Cancer Center and performed in accordance with approved guidelines and regulations.

### Immunohistochemistry and immunoblotting

The immunohistochemical staining for KCTD12 was performed in 157 paraffin-embedded primary CRC tissues as follows: the paraffin-embedded specimens were deparaffinized and stained with anti-KCTD12 antibody overnight. The KCTD12 expression was detected by the secondary-rabbit HRP-conjugated antibody and a DAB chromogen kit. The IHC scores for KCTD12 in CRC tissues were calculated by two independent pathologists and were composed of the score for the percentage of positively stained tumor cells and the grade of the staining intensity. The percentages of positively stained tumor cells were scored according to the following rules: 0, no positive tumor cells; 100, <25%; 200, 25%–50%; 300, >75%. The staining intensities were divided into no staining, weak staining, moderate staining and strong staining. The expression of KCTD12 in adjacent carcinoma and malignant tissues were compared in accordance with the above rules.

Protein extractions form the CRC cells and tissues were subjected to SDS-PAGE, followed by transfer onto PVDF membranes. The membranes were incubated with the antibodies anti-KCTD12, anti-CD44, anti-CD29, and anti-CD133 (Proteintech, Chicago, IL, USA). The Hsp70 and GAPDH antibodies (SantaCruz, Santa Cruz, CA) were used as controls.

### Cell proliferation assays

For the proliferation assay, HT29, DLD1 and HCT116 cells were seeded at a density of 1.5 × 10^3^ cells per well in 96 well microplates and cultured for six days. For cell viability analysis, the density of cells was 5 × 10^3^ cells per well in 96-well microplates. One day following seeding, cells were stained with 3-(4,5-dimethyl-2thiazolyl)-2,5-diphenyl-2H-tetrazolium bromide (MTT) dye (0.5 mg/ml, Sigma, Saint Louis, MI) for 4 h at 37 °C, the culture medium was removed, and cells were dissolved in dimethyl sulphoxide (DMSO, Sigma, Saint Louis, MI). The absorbance was measured with a multifunctional microplate reader at 490 nm. The absorbance was normalized to the absorbance at the first day and calculated. Each experiment was performed in triplicate.

### RNA Isolation and qRT-PCR

Total RNA was extracted from cells using the RNA extraction kit (Qiagen, Hilden, Germany) according to the manufacturer’s instructions. The RNA concentration was measured with a NanoDrop ND-1000 spectrophotometer (NanoDrop Technologies, Rockland, DE, USA). Reverse transcription and qRT-PCR was performed with the SYBR GreenER^TM^ two step qPCR kit (Invitrogen, Paisley, UK).

### Stable cell lines

To establish stable cell lines with KCTD12 knockdown or overexpression, we selected two effective sgRNA sequences for KCTD12 knockdown with CRISPR (Clustered Regularly Interspaced Short Palindromic Repeats) technology^32^. Stable cell lines with overexpression of KCTD12 were established based on Pbabe-retrovirus vectors. 293T cells were co-transfected with expression vectors and virus skeleton vectors. Viruses were collected after incubation for 24 h and 48 h and were then used to infect HT29, DLD1 and HCT116 cells. The stable cell lines were then selected with 0.5 μg/ml puromycin and isolated after10 days incubation.

### Flow cytometry analysis

For the apoptosis assay, the cells were harvested and washed with cold PBS, and then the cells were stained first with Annexin V (Nanjing kaiji Bio-Tek Corporation, Jiangsu, China) for 20 min at 4 °C in the dark and second with PI solution (50 μg/ml). Cells were then analyzed using a CytomicsTM FC 500 instrument (Beckman Coulter, USA). The results were analyzed and displayed with CXP software and Flow J software.

For the assay of CD molecules expression, the cells (5 × 10^5^) were collected and washed twice with PBS. The cells were stained with 2 μg anti-CD44 and anti-CD133 antibodies or respective control IgG in the dark and analyzed with the equipment. The analyses were performed with Flow J software. Each experiment was performed in triplicate.

### Colony formation assays

Cells were seeded in 6-well plates at a density of 5 × 10^2^ cells per well and cultured for 12 days. The colonies were washed once with PBS and fixed with methyl alcohol for 30 min at room temperature. The colonies were stained with 1% crystal violet for 2 min and were counted.

### Sphere formation assays

Five thousand cells per well were seeded in ultra-low attachment 6-well plates (Corning, Tweksburg, MA) and incubated in DMEM/F12 (1:1) supplemented with B27 (Invitrogen, Life Technologies Inc. Grand Island, NY), 25 ng/ml fibroblast growth factor-basic (bFGF, Sigma, St, Louis, MO) and 20 ng/ml epidermal growth factor (EGF, Sigma, St, Louis, MO). The number of spheres with a diameter of >50 μm was quantified by Image J software.

### *In vivo* tumorigenicity experiments

Male BALB/c nude mice (4 week old, 16–18 g) were randomly divided into 3 groups (n = 7/group) for the KCTD12 knockdown experiment and into 2 groups (n = 5/group) for the KCTD12 overexpression experiment. For tumor cell implantation, the cells with KCTD12 knockdown or KCTD12 overexpression (1.5 × 10^6^) suspended in 100 μl PBS were injected into the armpits of mice. The length, width and thickness of tumors were examined every two days, and the weights of tumors were calculated at the end of the experiment. All experiments were performed in accordance with the Institutional Animal Care and Use Committee of Sun Yat-sen University. All experimental protocol involving mice were approved by the ethical committee of Sun Yat-Sen University Cancer Center and performed in accordance with approved guidelines and regulations.

### The inhibitor for ERK1/2 and cell lines

HT29 cells were treated with 30 μM U0126 to inhibit the activity of the ERK1/2 signaling pathway of an equivalent concentration of DMSO as a control. The colorectal cancer cell lines HT-29, HCT116, and DLD-1 and the embryonic kidney cell line 293T were purchased from American Type Culture Collection.

### Cell viability assays

For cell viability analysis after treatment with imatinib and 5-Fluorouracil (5-FU), cells were plated in 96-well microplates at a density of 5 × 10^3^ cells per well and cultured overnight; This was followed by the addition of increasing concentrations of drugs and incubation for 24 h or 48 h, and then cell viability was determined by the MTT assay. For cell apoptosis analysis, cells were seeded in 6-well plates at a density of 5 × 10^5^ cells per well and treated with 100 μM imatinib for 24 h or with 10 μg/ml 5-FU for 48 h. The apoptosis rates were detected with the Annexin V/PI kit according to the manufacturer’s instructions.

### SP cells assay

The effects of KCTD12 on the SP cells fraction were evaluated using KCTD12 knockdown HT29 cells; 7 × 10^5^ cells were collected and divided into two groups. One group was pretreated with 50 μg/ml verapamil for 15 min at 37 °C, and then both groups were incubated with the DNA binding dye Hoechest33342 at a final concentration of 0.1 μg/ml for 90 min at 37 °C with gentle agitation every 15 min.

### Statistical analysis

Statistical analyses were performed with the SPSS version 16.0 software (version 16.0, SPSS Inc., Chicago, IL, USA) and the GraphPad PRISM software (GraphPad Software Inc., San Diego, CA). The correlations between KCTD12 expression and OS and DFS were analyzed with Kaplan-Meier Survival and the log rank test. The relationship between KCTD12 expression and clinicopathological features of CRC cancers was determined by the Pearson Chi-Square test. For multivariate statistical analysis, a Cox regression model was used. Data were analyzed using Student’s *t*-test or one/two way ANOV methods and represented as the means ± SEM; *p* < 0.05 was considered statistically significant.

## Additional Information

**How to cite this article**: Li, L. *et al*. KCTD12 Regulates Colorectal Cancer Stemness through ERK Pathway. *Sci. Rep*. **6**, 20460; doi: 10.1038/srep20460 (2016).

## Supplementary Material

Supplementary Information

Supplementary figures

## Figures and Tables

**Table 1:** Correlation between KCTD12 expression and clinicopathologic characteristics of colorectal cancer patients.

Characteristics	n	KCTD12 expression		Chi-square test	
		Low or none No. cases (%)	High No. cases (%)	p value	χ^2^
**Gender**					
Male	89	72	17	0.679	0.225
Female	68	57	11		
**Age**					
≤55	53	44	9	0.040	1.000
>55	104	85	19		
**Clinical Stage**				0.027*	**5.174**
I+II	50	36	14		
III+IV	107	93	14		
**T classification**				0.253	1.745
T1+T2	24	22	2		
T3+T4	133	107	26		
**N classification**				0.129	2.764
N0	57	43	14		
N1 +N2+ N3	100	86	14		
**M classification**					
M0	120	95	25	0.089	3.125
M1	37	34	3		
**Pathologic Differentiation**					
Well	17	14	3	0.053	0.974
Moderately	125	103	22		
Poorly	15	12	3		
**Position**				0.399	11.546
colon	128	114	14		
rectal	29	18	11		
**Vital status (at follow-up)**				0.003*	**11.513**
Alive	83	62	21		
Death	74	67	7		
**Tumor size (cm)**				0.021*	**5.722**
≤5	113	98	15		
>5	44	31	13		

^*^P  <  0.05.

**Table 2:** Univariate and multivariate analysis for overall survival (Cox proportional hazards regression model).

Variable	Univariate analysis		Mutivariate analysis		
	No.	*p*value	HR	95% CI	*p*value
**KCTD12 expression**		0.001*	0.239	0.084–0.677	0.007*
low expression	129				
high expression	28				
**Age**		0.253	1.139	0.678–1.914	0.622
≤55	53				
>55	104				
**Gender**		0.537	0.867	0.514–1.462	0.592
Male	68				
Female	89				
**Tumor size (cm)**		0.965	1.814	1.016–3.239	0.044*
≤5	113				
>5	44				
**Clinical Stage**		0.000*	2.404	0.829–6.972	0.106
I+II	50				
III+IV	107				
**T classification**		0.191	1.035	0.476–2.250	0.932
T1+T2	24				
T3+T4	133				
**N classification**		0.000*	1.442	0.897–2.318	0.131
N0	57				
N1	46				
N2+N3	54				
**M classification**		0.000*	2.289	1.320–3.967	0.003*
M0	121				
M1	36				
HR: Hazard Ratio; CI, confidence interval; *p < 0.05					

^*^ *P* < 0.05.
