# Supplementary material for: KCTD12 Regulates Colorectal Cancer Cell Stemness through the ERK Pathway
Source: Sci Rep. 2016 Feb 5;6:20460. doi: 10.1038/srep20460 (PMC4742820; doi:10.1038/srep20460)
Supplement: Supplementary Information [file srep20460-s1.doc]

KCTD12 Regulates Colorectal Cancer Cell Stemness through the ERK Pathway

Liping Li ^1#^, Tingmei Duan ^1#^, Xin Wang ^1#^, Ru-Hua Zhang^1^, Meifang Zhang^1^, Suihai Wang^2^, Fen Wang^3^, Yuanzhong Wu^1^, Haojie Huang^4^, Tiebang Kang^1^**^*^**

^1^State Key Laboratory of Oncology in South China, Sun Yat-Sen University Cancer Center, Guangzhou 510060, China.

^2^School of Biotechnology, Southern Medical University, Guangzhou 510515, China

^3^ Department of Pathology, First Affiliated Hospital of Sun Yat-sen University, Guangzhou 510080, China

^4^ Department of Biochemistry and Molecular Biology, Mayo Clinic College of Medicine, Rochester, MN 55905, USA

^#^These authors contributed equally to this work.

**^*^ Correspondence to**:

Dr. Tiebang Kang, Tel: 86-20-8734-3183. Fax: 86-20-8734-3170; E-mail: [kangtb@mail.sysu.edu.cn](mailto:kangtb@mail.sysu.edu.cn)

**Supplementary information**

**KCTD12 knock down sequences**

The two effective sgRNA sequences for KCTD12 knockdown: CGCTCGGGCTACATCACCAT; CAGCGACGTCTTTCCGCAAA with CRISPR (Clustered Regularly Interspaced Short Palindromic Repeats) technology. The lentiviral CRISPR/Cas9 vector was purchased from addgene (<http://www.addgene.org/>).

**Primers for qPCR**

| **Genes** | **F (5'-3')** | **R (5'-3')** | **size** |
| --- | --- | --- | --- |
| CD44 | CCCCCTTTCTTTTTCCAGTT | ACTTTCTGCCCCTCTCCACT | 201bp |
| CD133 | TTGTGGCAAATCACCAGGTA | TCAGATCTGTGAACGCCTTG | 162bp |
| CD29 | CCTACTTCTGCACGATGTGATG | CCTTTGCTACGGTTGGTTACATT | 128bp |
| KCTD1 | CAGAGGGACAGTCGGCC | GTATTTGGTGAGGGTGGCCAG | 174bp |
| KCTD2 | AACGTGGGAGGCACCTACTT | TGTCAATCAGATAGGCTCCTGTC | 139bp |
| KCTD3 | GAGATCGTCCAACTGAACGTAG | TTCTGCTTCATGCCTGAGAAC | 243bp |
| KCTD4 | CCTCTTCAGGCATGTCCTAAAC | TCTCTCTGGGTGTTAGCTGTT | 167bp |
| KCTD5 | GACACGGCAAGCTGGTGATTA | CGATGTTTTGCTGTCTCGTTCTC | 131bp |
| KCTD6 | ACGCAACTAACCATCACCACT | AGGTGGACCCTAAGAGAAACTTC | 161bp |
| KCTD7 | GACGCCGAAGACGACTTTCT | CGATGTTAAGGGGAACAACCTCA | 109bp |
| KCTD8 | CCGCTTCTACCTCAAGTTCACC | CTGAGGTGGTCGGAAG | 172bp |
| KCTD9 | AATGTTGGAGGGCGGTACTTT | ATGTGGGCCAGCATACTGTC | 205bp |
| KCTD10 | GGGTGGAGCCCTCTACTATAC | CACTGTCGGTGAGCACTTCC | 101bp |
| KCTD11 | CGGGGACCCCATCACTATGA | TCAGAGTCGGTGCAGAAAAGG | 77bp |
| KCTD12 | GCTCGGGCTACATCACCATCGG | GGGTCCCGGCTTTCGTTCAG | 160bp |
| KCTD13 | CCTGCACAACCGCAGTAACA | CCTGCCCGTAGAAAGACCAG | 170bp |
| KCTD14 | ATGTCTACTGTTGTGGAGCTGA | CCTTGGCTAAGCTAGAGAACATC | 115bp |
| KCTD15 | ATGGCACTGAACCCATCGTC | GGCTGGAGCTGATAGTAGCG | 175bp |
| KCTD16 | ATGGCTCTGAGTGGAAACTGT | TCAATGTGGAATGGCGAGTAAA | 130bp |
| KCTD17 | AGTCGGACCGGGATGAGAC | CCATGCCGGAGGAAGTTCA | 85bp |
| KCTD18 | AAGAGGTGCTAGATGTTCTCCG | TCCATCACGGTCAATAACACAAG | 164bp |
| KCTD19 | CTGTATGAGCAAGCATTGGGT | TCCATGTACGCCAGTAGTTCA | 145bp |
| KCTD20 | TGACAGTGACAGGTTATTGCG | AGGCATAGTCAAGTGAGAGGTC | 161bp |
| KCTD21 | GTGACGGCAAAGTGTTCCG | GTTGGCGTTGAAGACCTCCAT | 290bp |
| GAPDH | ACAGTCAGCCGCATCTTCTT | GACAAGCTTCCCGTTCTCAG | 259bp |
|  |  |  |  |
