# Supplementary material for: KCTD12 Regulates Colorectal Cancer Cell Stemness through the ERK Pathway
Source: Sci Rep. 2016 Feb 5;6:20460. doi: 10.1038/srep20460 (PMC4742820; doi:10.1038/srep20460)
Supplement: Supplementary figures [file srep20460-s2.doc]

Figure 1


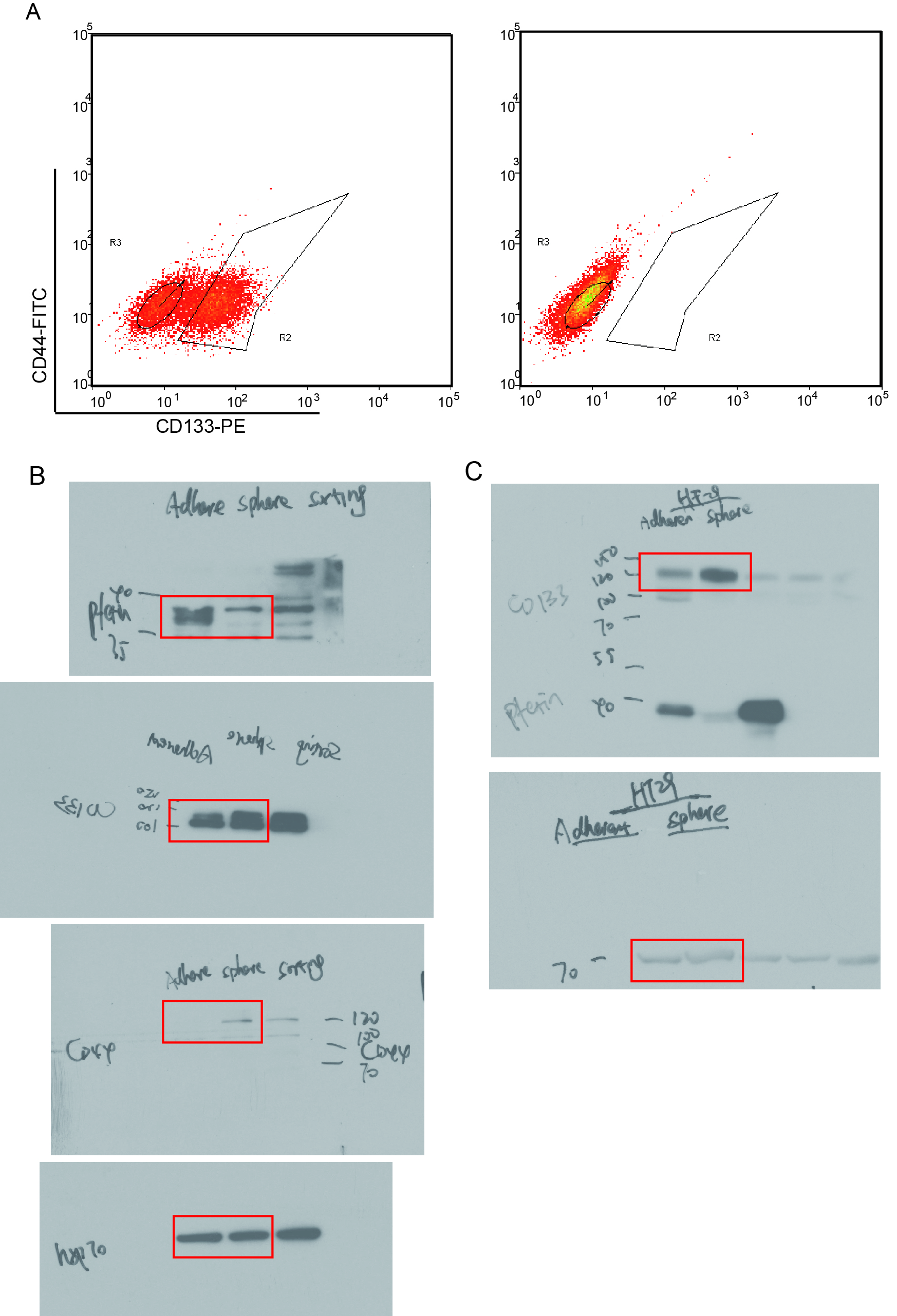


Supplementary Fig 1. KCTD12 is down-regulated in CSC-like HT29 cells.

1. Flow cytometric isolation of cancer stem cells based on CD133 and CD44 expression. FACS histogram showing CD133 and CD44 expression of spheroids in HT29 cells. The pattern of background CD133 and CD44 expression of adherent HT29 cells. The results showed the CD133 positive cells were sorted mainly, termed sorting group.
2. The western blotting results of KCTD12, CD133 and CD44 expression in spheroids, adherent cells and CD133/CD44 sorting HT29 cells.
3. The western blotting results of KCTD12 and CD133 expression in spheroids and adherent HT29 cells. These gels have been run under the same experimental conditions.

Figure 2


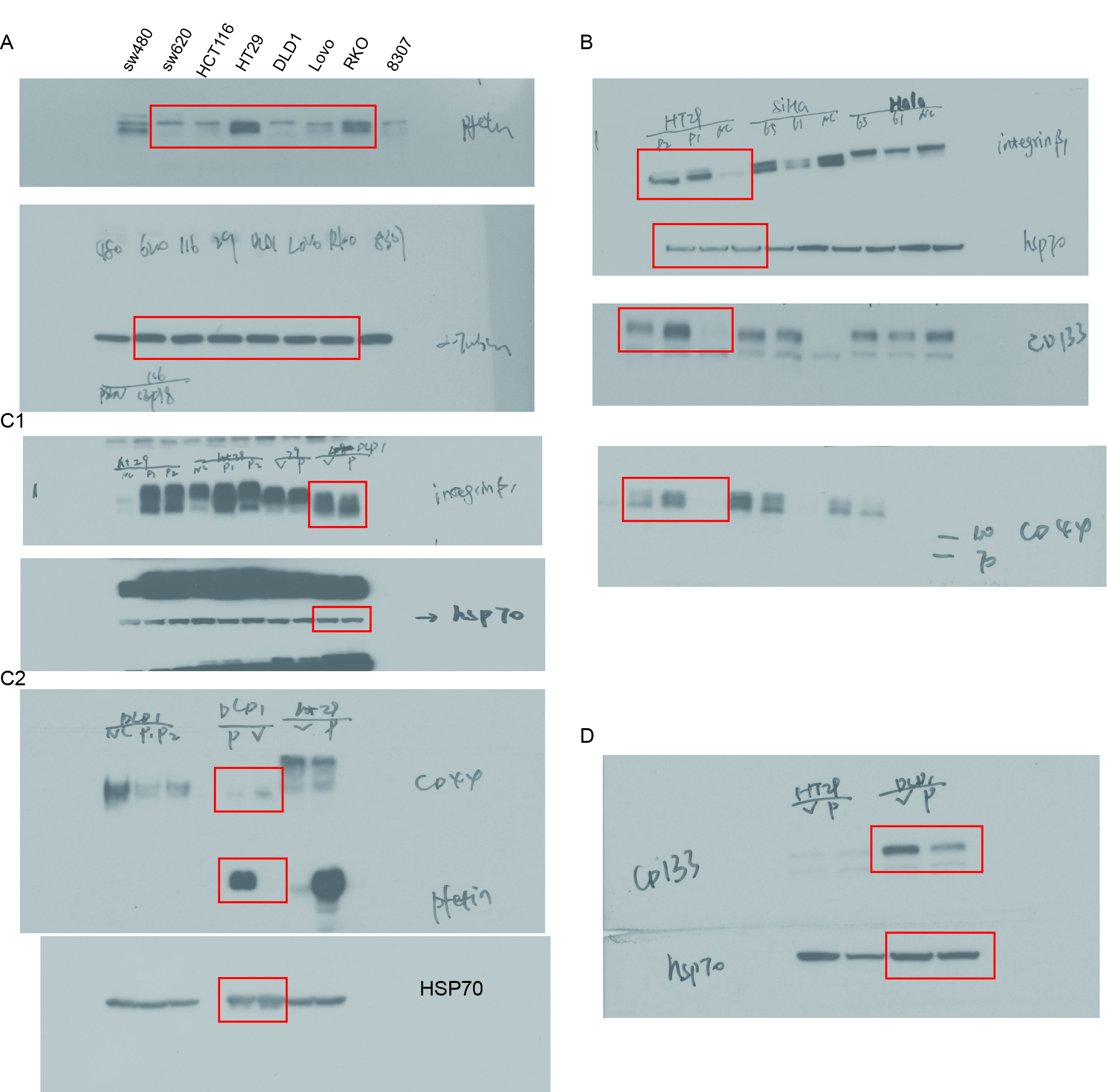


Supplementary Fig 2. KCTD12 suppresses the stemness of CRC cells.

1. The western blotting results of KCTD12 expression in several CRC cells. The gel has been run under the same experimental conditions.
2. The western blotting results of CD29, CD133 and CD44 expression in KCTD12 silencing HT29 cells. The gels have been run under the same experimental conditions.
3. The western blotting results of CD29 and CD44 expression in KCTD12 overexpressing DLD1 cells. The gel C1 and C2 have been run under the same experimental conditions respectively.
4. The western blotting results of CD133 in KCTD12 overexpressing DLD1 cells. The gel has been run under the same experimental conditions.

Figure 5


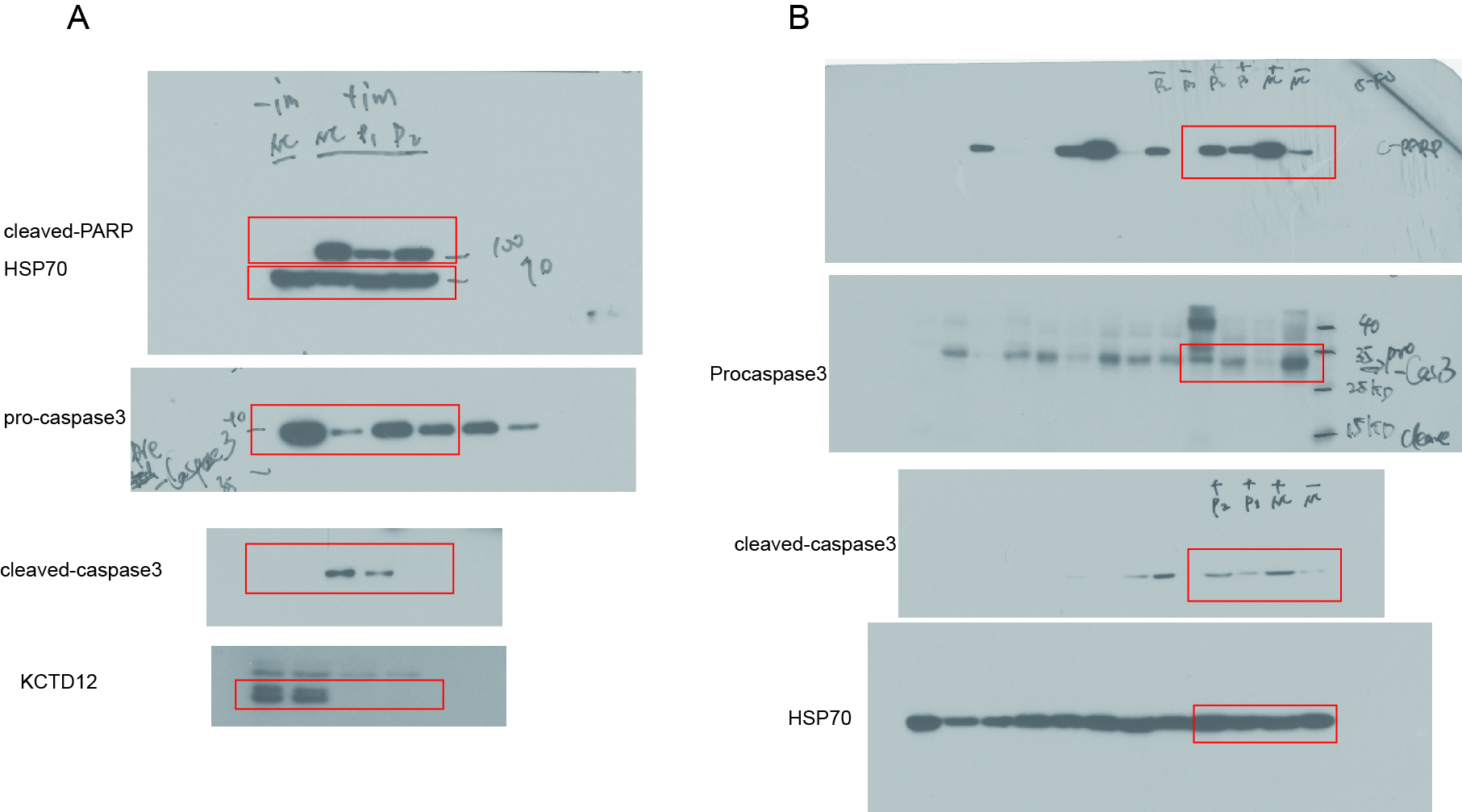


Supplementary Fig 5. Silencing of KCTD12 enhances the drug resistance.

1. The western blotting results of KCTD12, cleaved-PARP, pro-caspase3, cleaved-caspase3 and HSP70 expression under imatinib treatment. The gels have been run under the same experimental conditions. KCTD12 and cleaved-caspase3 were detected in the same gel, and pro-caspase3, cleaved PARP and HSP70 were detected in the other gel.
2. The western blotting results of cleaved-PARP, pro-caspase3, cleaved-caspase3 and HSP70 expression under 5-FU treatment.

Figure 6


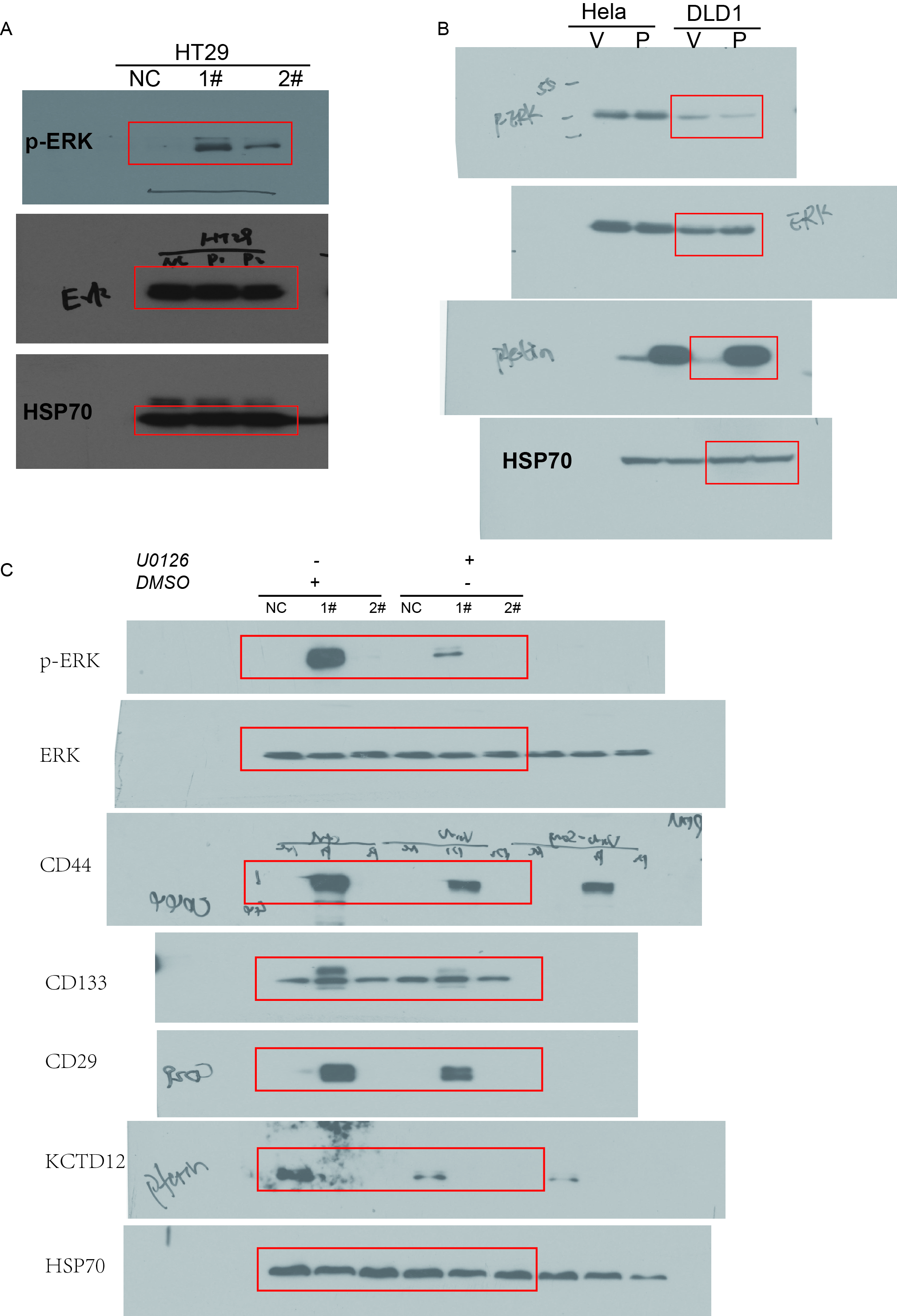


Supplementary Fig 6. KCTD12 regulates stemness of CRC cells via the ERK signaling pathway.

1. The western blotting results of p-ERK1/2, total-ERK1/2 and HSP70 expression in KCTD12 silencing HT29 cells, the gels were run under the same experimental conditions.
2. The western blotting results of p-ERK1/2, total-ERK1/2 and HSP70 expression in KCTD12 overexpressing DLD1 cells, the gels were run under the same experimental conditions.
3. The western blotting results of p-ERK1/2, total-ERK1/2, CD133, CD44, CD29 and HSP70 expression, the gels were run under the same experimental conditions.

Figure 7A


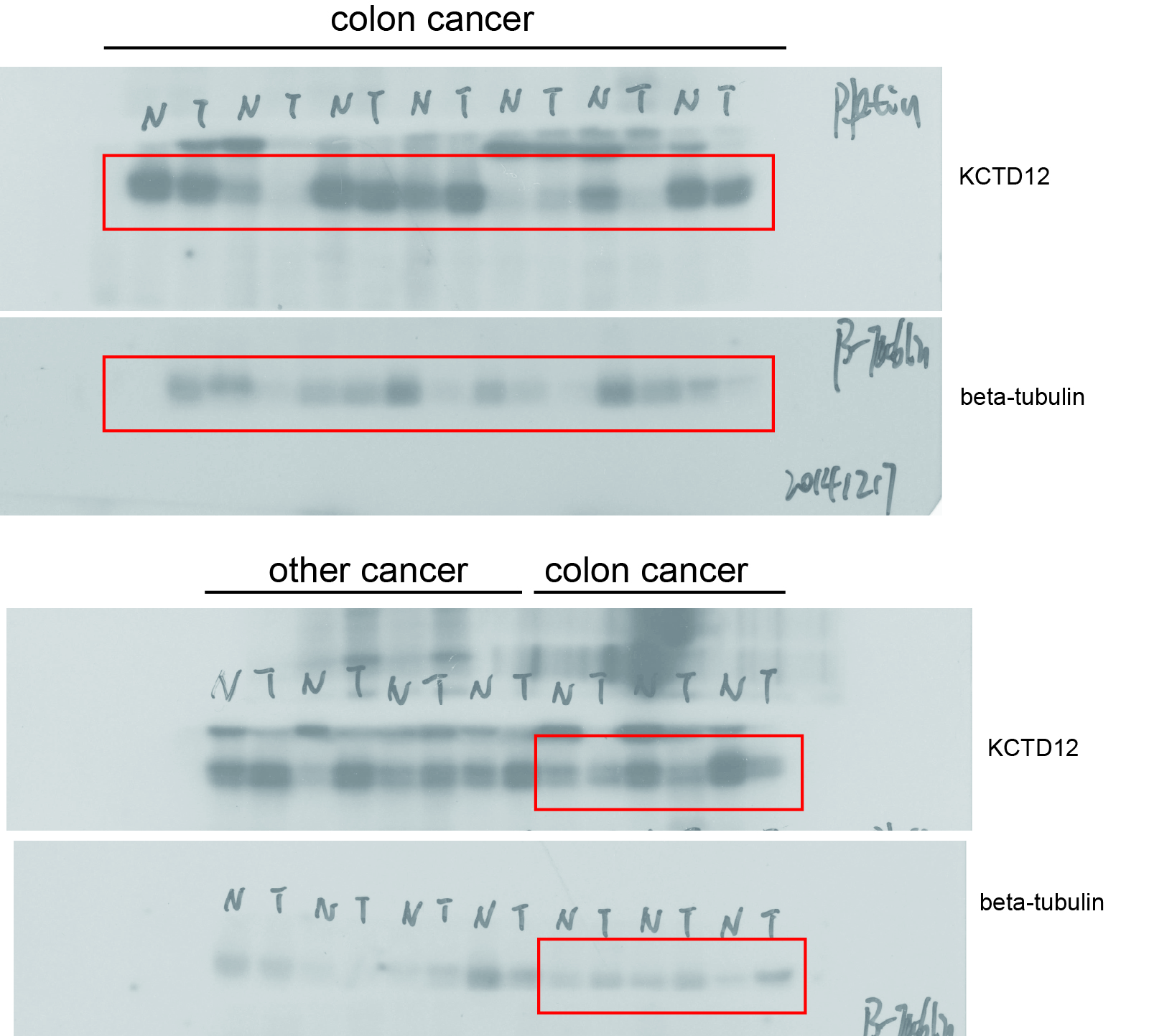


Supplementary Fig 7. KCTD12 regulates stemness of CRC cells via the ERK signaling pathway.

The western blotting results of KCTD12 and β-tubulin expression in CRC tissues were run under the same experimental conditions.
